# Employment and mental health in the working age population: a protocol for a systematic review of longitudinal studies

**DOI:** 10.1186/s13643-024-02613-1

**Published:** 2024-07-25

**Authors:** Fiona Aanesen, Rigmor C. Berg, Ingrid Løken Jørgensen, Benedicte Mohr, Karin Proper, Lars-Kristian Lunde

**Affiliations:** 1 National Institute of Occupational Health, PO Box 5330 Majorstuen, Oslo, 0304 Norway; 2 Norwegian Institute of Public Health, PO Box 222 Skøyen, Oslo, 0213 Norway; 3 Dutch National Institute for Public Health and the Environment, PO Box 1, 3720 BA Bilthoven, The Netherlands; 4 Department of Public and Occupational Health, Amsterdam University Medical Center, Amsterdam, The Netherlands

**Keywords:** Work, Re-employment, Employment transition, Unemployment, Depression, Anxiety, Psychological distress, Systematic review, Meta-analysis, Narrative synthesis

## Abstract

**Background:**

Employment provides economic security, a social network, and is important for self-identity. A review published by van der Noordt and colleagues in 2014 showed that employment was beneficial for depression and general mental health. However, an updated synthesis including research published in the last decade is lacking. In the planned review, we aim to update, critically assess, and synthesise the current evidence of the association between paid employment (excluding precarious employment) and common mental health outcomes (depression, anxiety, and psychological distress) among the working age population in the labour force.

**Methods:**

We will follow recommended guidelines for conducting and reporting systematic reviews. Four electronic databases (MEDLINE, Embase, APA PsycINFO, and Web of Science) will be searched from 2012, using appropriate MeSH terms and text words related to our inclusion criteria. We will screen the records against predefined eligibility criteria, first by title and abstract using the priority screening function in EPPI-Reviewer, before proceeding to full-text screening. Only studies investigating the longitudinal relationship between employment and common mental health outcomes will be included. We will search for grey literature in OpenAlex and conduct backward and forward citation searches of included studies. The methodological quality of the included studies will be assessed using the Cochrane risk-of-bias tool (RoB 2), Risk Of Bias In Non-randomised Studies of Interventions (ROBINS-I), or the Newcastle–Ottawa scale (NOS). We will conduct a narrative review and, if possible following pre-set criteria, conduct random-effects meta-analyses to estimate the pooled effect of employment on depression, anxiety, and psychological distress, across the included studies.

**Discussion:**

An updated review of the association between non-precarious employment and mental health outcomes is needed. In the planned review, we will assess the quality of the included studies and synthesise the results across studies to make them easily accessible to policy makers and researchers. The results from the review can be used to aid in policy decisions and guide future research priorities.

**Systematic review registration:**

PROSPERO CRD42023405919.

**Supplementary Information:**

The online version contains supplementary material available at 10.1186/s13643-024-02613-1.

## Background

Work is an important part of people’s life and central to self-identity and well-being [1]. Several systematic reviews have shown that employment is associated with positive mental health outcomes [2–4]. In 2014, van der Noordt and colleagues reviewed the literature on health effects of employment published from 1990 to 2012. The review included 33 prospective observational studies comparing the health effects of all types of employment to unemployment. The pooled results showed that employment was a protective factor for depression (odds ratio (OR) 0.52, 95% confidence interval (CI) 0.33 to 0.83) and psychological distress (*OR* 0.79, 95% *CI* 0.72 to 0.86) [2]. Studies with varying methodological quality and various employment conditions were included in the meta-analyses. Due to large heterogeneity among studies, the authors recommended caution in the interpretation of the results [2]. Furthermore, the possibility for causal inference was hampered by ‘the healthy worker effect’, a common risk of bias (RoB) in occupational epidemiology studies [5]. Healthy individuals are both more likely to gain employment and to remain employed over time, whilst people with poor health are more likely to leave the workforce. These selection effects may lead to overestimation of the health effects of employment in observational studies [5]. A randomised controlled trial (RCT) is the best study design to control for confounding and to study causal effects. However, RCTs are rarely conducted to study the effect of employment due to ethical and feasibility reasons. During the last decade, the possibility of using advanced statistical models for causal inference has increased due to large improvements in computer performance. Statistical models including propensity score matching, difference in difference, and fixed effects regression have been used to reduce problems of endogeneity in studies investigating health effects of employment [6–9].

**Fig. 1:**
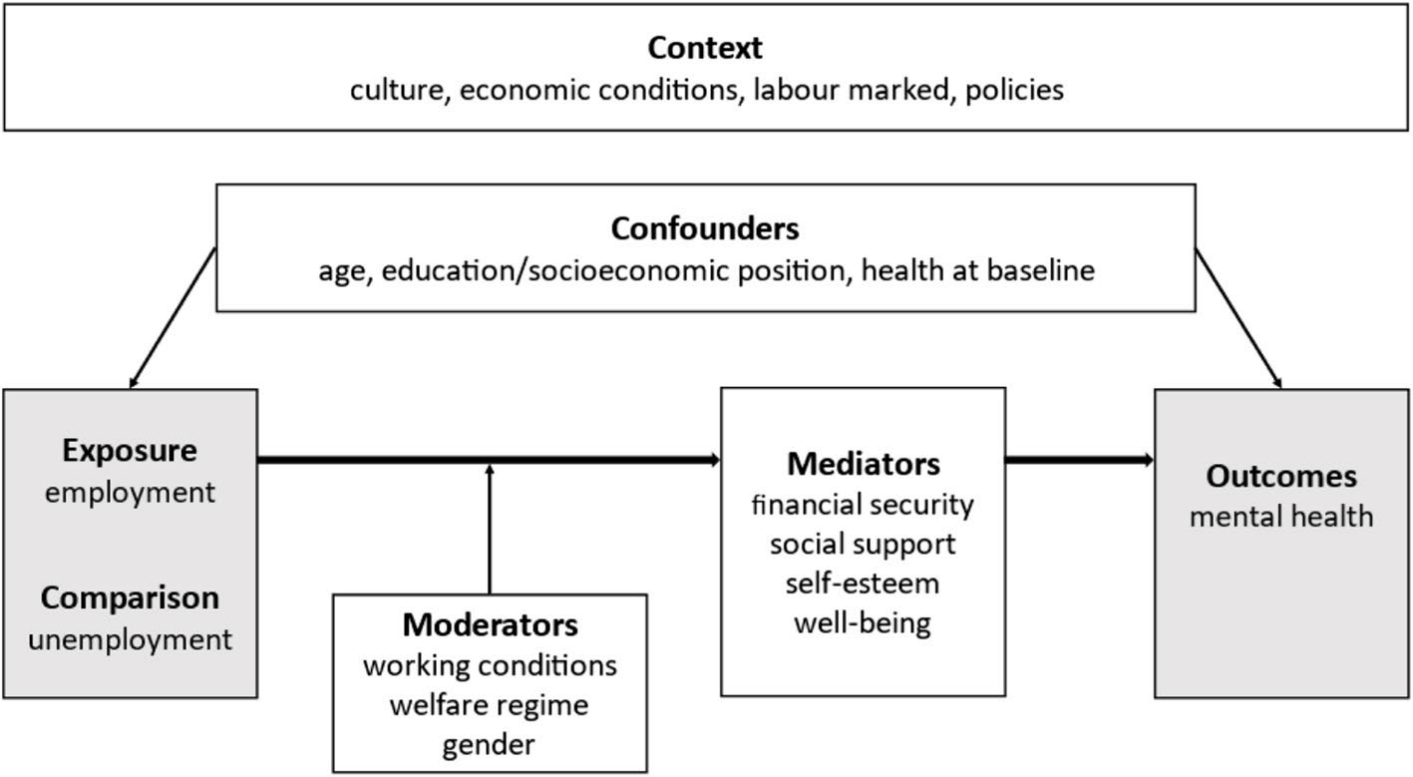
Logic model showing possible relationships between employment and mental health (depression, anxiety, and psychological distress)

Employment provides financial security, social purpose, and social support [10], factors that are beneficial for self-esteem and wellbeing. Unemployment on the other hand is associated with negative mental health outcomes [11–13]. However, welfare regimes could moderate the negative effects of unemployment [14, 15]. The Nordic social democratic welfare regimes are characterised by universalism, generous benefits, and extensive welfare services compared to welfare regimes in other countries [16]. The social protection provided in the Nordic countries reduces the financial hardship experienced by the unemployed [17]. Furthermore, several studies have shown that the effect of unemployment on health is stronger for men compared to women [6, 9, 11], which could be explained by social norms and gender roles [9, 11]. Work can have both detrimental and positive effects on mental health [18], depending on the working conditions [19]. Recent reviews have shown that people with stable working conditions have better self-rated health and less mental health symptoms, compared to workers in precarious employment [20, 21]. Figure 1 shows possible mechanisms of how employment and unemployment could affect mental health, including possible confounders, mediators, and moderators.

Several recent reviews have synthesised the literature on the health effects of precarious employment [20–22]. However, to our knowledge, results from longitudinal studies published after 2012, investigating the mental health effects of non-precarious employment in the general working population, have not been reviewed. Several observational studies published after 2012 have used statistical models to increase the possibility for causal inference [6–9]. The aim of the planned systematic review is to update the review conducted by van der Noordt and colleagues. The objective is to systematically review and synthesise the evidence regarding the effect of employment on mental health among the general working age population. To focus the scope of the review and facilitate data synthesis, we will include outcomes measuring depression, anxiety, and psychological distress and exclude studies focusing solely on precarious employment. The objective of the present protocol is to describe the methods we will use to answer the review question: ‘Is working in non-precarious paid employment, prospectively associated with common mental health outcomes (depression, anxiety, and psychological distress) in the general working age population?’ Additionally, to investigate if *welfare regime (Nordic social democratic vs. other types of welfare regimes), or gender (women vs. men)* moderate possible associations between working in paid employment and common mental health outcomes.

## Methods

Our review protocol adheres to the Preferred Reporting Items for Systematic review and Meta-Analysis Protocols (PRISMA-P) 2015 statement [23]. A PRISMA-P checklist for this protocol is provided in Additional file 1. The protocol was registered in the International Prospective Register of Systematic Reviews (PROSPERO) in April 2023, identification number CRD42023405919. The review will be conducted in line with the *Cochrane Handbook for Systematic Reviews of Interventions* [24] and the guidance for Conducting Systematic Reviews and Meta-Analyses of Observational Studies of Etiology (COSMOS-E) [25]. The review will be reported in accordance with the PRISMA 2020 guideline for reporting systematic reviews [26] and the proposal for reporting of Meta-analysis Of Observational Studies in Epidemiology (MOOSE) [27]. Any modifications of the methods described in the present protocol will be reported in PROSPERO and in the published review.

### Eligibility criteria

The review will include studies investigating the relationship between paid employment and mental health. An overview of the inclusion and exclusion criteria is provided in Table 1. Studies not providing information concerning our exclusion criteria, and studies that report outcomes separately for eligible participants, will be included.

**Table 1:** Eligibility criteria

Domain	Inclusion criteria	Exclusion criteria
Population	Age ≥ 15 In the general labour force	Selected groups not representative of the general working population, e.g.: • Students • Pensioners • Individuals with intellectual disability • Migrants/immigrants
Intervention/exposure	• Paid employment • Transition from unemployment to paid employment	• Vocational training programmes/sheltered employment/set-aside jobs for people with disabilities • Return-to-work interventions • Precarious employment (insecure/temporary, informal employment) • Exit from work • School to work transitions • Bridge employment (paid work after retirement) • Voluntary work
Comparison	Unemployment	• Not in labour force • Sickness absence • Disability pension • Retirement
Outcomes	• Depression • Anxiety • Psychological distress	• Infections/communicable diseases • Biomarkers • Mortality • Diagnoses other than depression or anxiety • Sickness absence • Disability pension • Substance abuse/smoking • Health behaviours • Social network/social support • Self-esteem • Resilience • Life satisfaction • Sense of coherence • Well-being • Sleep • Medication
Studies	Longitudinal studies: • Randomised controlled trials • Cohort • Panel • Case crossover • Non-randomised controlled trials • Interrupted time series	• Other study designs (e.g. case control, cross-sectional, time series, qualitative, Delphi) • Discussion papers • Essays • Book chapters • Systematic reviews and meta-analyses

We will include studies meeting the following criteria:

- Population

Studies including individuals in the labour force, aged 15 years or older. Fifteen is the legal age for full-time employment, set by the International Labour Organization (ILO) [28]. Studies from any country and setting are eligible. Exclusion criteria are as follows: selected groups not representative of the general working population, e.g. migrants/immigrants, individuals with intellectual disabilities, students or pensioners (working in addition to studying or receiving pension).

b) Intervention/exposure

We will include studies describing part-time or full-time paid employment in any occupation or setting, including employees and self-employed workers. Furthermore, we will include studies investigating the effect of transition from unemployment to part-time or full-time paid employment. Studies using any measures of employment status will be included (self-reported, workplace reported, or registry data). Intervention studies where employment is the intervention will be included. We will exclude studies investigating the effect of return-to-work interventions, studies examining the health effects of exit from work (transitioning from employment to unemployment, disability pension, sickness absence, or retirement) or transitions from school to work. We will also exclude studies if more than 50% of the workers are working outside the competitive labour force (e.g. vocational training programmes, unpaid trainee work, sheltered employment or voluntary work) or in precarious employment.

c) Comparison

The comparison will be participants who are unemployed and in the general labour force. Studies including any measures of unemployment will be included (self-reported, workplace reported, or registry data). We define the unemployed as all persons aged at least 15 years, who are without work and are currently seeking work (except selected groups not representative of the general labour force, e.g. immigrants/migrants, individuals with intellectual disabilities, students, and pensioners). We will exclude studies comparing workers to individuals not working due to sickness absence, disability, or retirement.

d) Outcomes

Studies including at least one of the following mental health outcomes will be included: *depression*, *anxiety*, or *psychological distress*. The outcomes can be measured through clinical diagnoses and self-reported or interview administrated generic validated questionnaires, e.g. the Patient Health Questionnaire (PHQ), the Centre for Epidemiological Studies-Depression (CES-D), the Beck Depression Inventory (BDI), the Hopkins Symptoms Checklist (HSCL), the Generalized Anxiety Disorder scale (GAD), the General Health Questionnaire (GHQ), Distress Questionnaire (DQ) and the Mental Health Inventory (MHI). We will exclude measurement tools that are developed specifically for certain types of depression or anxiety (e.g. postpartum depression or agoraphobia).

e) Study designs

We will include quantitative longitudinal studies (with data collection from the same participants at minimum two different time points), investigating the relationship between employment and the eligible mental health outcomes (described in section d). To be included, the studies must measure employment status at a timepoint prior to the measurement of the mental health outcome. Eligible study designs include randomised controlled trials (RCTs), non-randomised controlled trials (N-RCTs) and interrupted time series, cohort studies, panel studies and case-crossover designs. Mixed-method studies are eligible if they include longitudinal quantitative data; all other study designs will be excluded. We will exclude studies that lack a comparison group of unemployed persons; however, participants may act as own controls.

We will include papers written in languages understood by the review team: English, French, German, Dutch, Spanish, Finnish, Italian, or Scandinavian languages. Papers written in other languages will not be included. We will include all publication formats with a full text that describes the methods and results of the study (articles, reports, theses, etc.).

### Information sources and search strategy

A research librarian at the National Institute of Occupational health will develop and pilot a search strategy in collaboration with the first author and a research librarian from the Norwegian Institute of Public Health. The search includes Medical Subject Headings (MeSH) terms and text words related to employment and unemployment (e.g. work, employment, re-employment, unemployment) and mental health outcomes (e.g., depression, anxiety, psychological distress).

#### Database searches

A research librarian will search the following four electronic databases from 2012: MEDLINE (Ovid), Embase, APA PsycINFO, and Web of Science Core Collection. The search strategy for MEDLINE is presented in Appendix 1. Search strategies will be tailored for the other databases and made available in the published review.

#### Other sources

We will search reference lists for eligible studies from previous systematic reviews, meta-analyses, and from the included studies. Electronic cited reference searches of included studies and searches for grey literature will be conducted through OpenAlex. OpenAlex is a large open catalogue of scholarly work, including grey literature hosted in different venues [29]. Studies included in the review by van der Noordt et al. [2] meeting our inclusion criteria will be included in the current review.

### Study selection

The search records will be uploaded to EndNote [30], and duplicates will be removed. We will use the EPPI-Reviewer software [31] for study selection. First, we will screen titles and abstracts. During this process, we will use the priority screening function in EPPI-Reviewer. This machine learning function will be used to order relevant records at the top of the screening list*.* After including at least five records, priority screening predicts a model that presents records in order from highest to lowest likelihood of meeting the inclusion criteria. The model is updated after every 25 records screened, and the accuracy of the predictions gradually increases throughout the screening process. To ensure consistency, pilot screening of the first 100 records will be caried out by the entire research team. After this, the references will be independently screened for eligibility by two reviewers against prespecified inclusion and exclusion criteria (Table 1). Frequent consensus meetings will be held to solve disagreements through discussions. When we have screened 200 consecutive records that do not meet our inclusion criteria, we will switch to single screening. After screening a further 400 consecutive records that do not meet our inclusion criteria, we will stop screening.

After screening of titles and abstracts, we will proceed to full-text screening of relevant records to decide final inclusion. Inclusion will be decided through consensus. Disagreements will be solved through discussion and, if necessary, by a third reviewer. Reasons for excluding publications read in full text will be described in the review or in the supplementary material. The number of records from the searches and the selection process will be depicted in a PRISMA flow diagram.

### Data extraction

We will develop a data extraction sheet in Microsoft Excel [32]. The sheet will be piloted by all reviewers involved in the data extraction, and necessary modifications will be made until consensus is reached. Data extraction will be conducted by one reviewer and checked by a second reviewer. If needed, we will contact study authors for missing data. The information to be extracted from the studies is shown in Table 2.

**Table 2:** Data extraction from the included studies

Domain	Data to be extracted
*Study characteristics*	Title, first author, publication year, study design, study setting (location and country), sampling strategies, sample size, data collection period (dates), follow-up time (length to each follow-up), attrition at each time point
*Participant characteristics*	Age, gender, education level, socioeconomic status, civil status, and occupation. Employment status (length of employment, numbers working full-time, part-time, and unemployed). Mental health outcomes at baseline
*Intervention/exposure*	Definition of employment or employment trajectories used in the study. Measurement tool used to measure employment or employment trajectories or description of employment intervention
*Comparison*	Definition of unemployment used in the study and measurement tool used to measure unemployment
*Outcomes*	Type of mental health outcome, measurement tool/instrument (including upper and lower limits, score interpretation, and definition of thresholds), timing of outcome measurement
*Statistical analyses*	Number of participants in each group for every analysis (employed vs. unemployed), description of analysis methods, covariate(s) included in analyses, methods used to address missing data
*Results*	Detailed numerical data for eligible mental health outcomes in each group including the following: 2 × 2 tables, means with standard deviations, and effect estimates, e.g. odds ratio, risk ratio, regression coefficient, mean difference, standardised mean difference with confidence intervals, standard errors, *p*-values

We will extract data for the three main outcomes: depression, anxiety, and psychological distress. If several analyses have been conducted for the same outcome, we will extract data for the unadjusted analysis and for the analysis that closest resemble the following model: effect of employment/re-employment compared to unemployment on one of the included mental health outcomes, adjusted for baseline values of the mental health outcome, age, gender, education, and/or socioeconomic position.

### Methodological quality assessment of the included studies

To identify selective reporting of outcomes and post hoc analyses, we will investigate available protocols and registrations of studies. We will also look for signs of post hoc decisions regarding analyses including differences between variables assessed in questionnaires and those reported in the papers, data-driven cut-off points, or unjustified subgroup analyses.

Two authors will independently assess the methodological quality of the included studies. If several papers report findings from the same study, they will be assessed together. To ensure consistency, we will pilot the quality assessment among the reviewers. During the pilot stage, every reviewer will assess one study of each study design included in the review. Any uncertainty in judgements will be discussed, and ambiguity was resolved during a consensus meeting.

#### Quality assessment tools

We will use the second version of the Cochrane risk-of-bias tool for randomised trials (RoB 2) [33]. The tool includes assessment of risk of bias (RoB) in five domains: (1) *the randomisation process*, (2) *deviations from intended intervention*, (3) *missing outcome data*, (4) *outcome measurement*, and (5) *selection of reported result*. A judgement of either low RoB, some concerns, or high RoB is made for each domain based on answers to several signalling questions [34]. To assess RoB in N-RCTs, we will use the Risk Of Bias In Non-randomised studies of Interventions (ROBINS-I) tool [35]. The ROBINS-I tool is used to assess RoB against a hypothetical ideal target trial. The tool includes judgements of RoB in seven domains: (1) *confounding*, (2) *selection of participants*, (3) *classification of interventions*, (4) *deviations from the intended intervention*, (5) *missing data*, (6) *measurement of outcomes*, and (7) *selection of the reported results* [35]. A judgement of either low, moderate, serious, or critical RoB or no information is given for each domain. Finally, the Newcastle–Ottawa scale (NOS) [36] will be used to assess the methodological quality of observational studies. The NOS includes judgements in three domains: *(1) selection of study groups*, *(2) comparability of the groups, and (3) the attainment of the exposure or outcome of interest*. A star system is used to indicate overall methodological quality of the study.

### Data synthesis

#### Narrative synthesis

The review will include studies from various settings, with different study designs, populations, follow-up periods, outcomes, outcome measures, and adjustment for a variety of confounding factors. Furthermore, employment and unemployment conditions vary greatly between countries. Therefore, we anticipate that there will be large heterogeneity between the studies included in the review and will conduct a narrative synthesis. We will follow the reporting guideline for Synthesis Without Meta-analysis (SWiM) in systematic reviews [37].

Study characteristics and results of the included studies will be presented in tables together with the methodological quality assessment. Study information will include name of the fist author, year of publication, study design and setting, data collection period, timing and number of waves, participant characteristics, description of exposure/intervention and comparison, outcome measures and cut-off points, numbers included in the analyses, type of statistical analysis, and covariates. To aid comparison of results across studies, effect estimates will be reported as either OR or standardised mean difference (SMD) with 95% CIs. We will use the Review Manager (RevMan) calculator for effect transformation [38] and follow the guidance provided in the *Cochrane Handbook for Systematic Reviews of Interventions* [24]. If it is not possible to calculate OR or SMD, we will provide the effect estimate reported in the original study. We will present one table with studies investigating transitions from unemployment to employment and a separate table for studies comparing employment to unemployment.

The logic model (Fig. 1) including the possible moderators, and how well the study controls for ‘the healthy worker effect’, will be used as a guide for grouping the studies for the narrative synthesis. We will illustrate the results from the included studies in appropriate plots depending on the available information (e.g. forest, box and whisker, bubble, albatross, harvest [39]).

### *Meta*-analyses

If two or more similar studies report comparable outcomes, we will conduct meta-analyses to estimate the combined effect across the included studies. The results of the meta-analyses will be presented in forest plots showing effect estimates for each study, with 95% CIs and methodological quality assessment including combined estimates of average intervention effects across studies, with 95% CIs. If several papers report results from a single study, only results from one of the papers will be included in each meta-analysis. Separate meta-analyses will be conducted for observational studies and for RCTs. Because we anticipate there to be heterogeneity between the studies, we will conduct random-effects meta-analyses that incorporate an assumption that the studies are estimating related but different intervention effects [40]. The analyses will be conducted by two of the study authors in cooperation with a statistician. We will not conduct meta-analyses if there is considerable statistical or methodological heterogeneity between studies [40]. Heterogeneity will be assessed by comparing study characteristics, inspecting forest plots of effect estimates and confidence intervals, and with *τ*^2^ and the Higgins’ *I*^2^ statistic [40].

We will conduct separate meta-analyses for studies investigating the effects of re-employment and for studies investigating the effects of employment. We will include studies using any employment definition meeting the inclusion criteria listed in Table 1. If possible, we will conduct separate analyses for each of the three main outcomes included in this review: depression, anxiety, and psychological distress. We will pool results from studies using different outcome measures. If studies measure the same outcome using different questionnaires, we will use SMD to pool continuous data and OR to pool dichotomous data. If different metrics are used to describe effect estimates across studies, we will transform them to mathematically comparable data. If the papers present results from several statistical models, we will include estimates from the model that controls for most of the following variables: baseline health, age, gender, education, and socioeconomic position. If a study includes multiple measures of the same outcome (e.g. different measures of depression), we will only include one outcome measure from the study in each meta-analysis. The following hierarchy will be used to decide inclusion: (1) the outcome judged as having lowest RoB and (2) the study’s primary outcome (used for the sample size calculations). If outcomes or effect estimates are incompletely reported and missing information is not possible to calculate or retrieve, the results will not be included in the meta-analyses.

#### Subgroup and sensitivity analyses

As described in the introduction, welfare regime and gender may moderate the effects of employment and unemployment on mental health outcomes. Based on the literature, we hypothesise that the mental health benefits of employment compared to unemployment will be stronger in countries with weak social welfare support compared to Nordic countries and stronger for men compared to women.

There are several methodological features that may lead to heterogeneity between the included studies. Employment contract and working hours could impact study results. Time between waves and length of employment could also be important. A recent study on the scarring effects of unemployment showed that it took on average 2 years for people to recover to their pre-job loss baseline values of mental health after re-employment [41]. Furthermore, studies with long intervals between waves could include unmeasured changes in employment status. Methodological quality could affect the study results, and studies that do not control for mental health at baseline may overestimate the positive effect of employment on mental health. Therefore, we plan to conduct the following subgroup and sensitivity analyses if there are adequate numbers of studies:

- A subgroup analysis by type of welfare regime: social democratic Nordic welfare regime (Nordic countries) vs. other types of welfare regimes
- A subgroup analysis by gender: women vs. men
- Subgroup analysis for studies with ≤ 2 years between waves vs. > 2 years
- Sensitivity analysis excluding studies that do not control for mental health at baseline
- Sensitivity analysis excluding studies with participants in part-time employment
- Sensitivity analysis including only studies with participants in stable employment
- Sensitivity analysis excluding studies with low-quality assessments

If inspections of study features and forest plots indicate that other subgroup or sensitivity analyses should be performed (e.g. by region, age group, study design, intervention, type of estimation model, type of employment, length of employment, or working conditions), we will identify these as post hoc exploratory analyses. Justifications for the decisions will be provided in the review.

### Publication *bias*

If more than 10 studies are included in any of the meta-analyses, we will construct funnel plots to visually assess possible small-study effects. Furthermore, we will conduct Egger’s regressions, to test the relationship between sample size and effect size. If the funnel plot and Egger’s test indicate small-study effects, we will investigate if this could be due to publication bias. If we suspect the results of the meta-analysis to be affected by publication bias, we will conduct sensitivity analyses excluding small studies.

### Reporting and dissemination

The results of the review will be reported in an article and submitted to a peer-reviewed journal. The results will also be disseminated on the website of the National Institute of Occupational Health in Norway. If there are any differences between the methods described in this protocol and the methods used whilst conducting the review, these will be reported in the published review.

## Discussion

The research published during the last decade concerning the mental health effects of non-precarious employment has not been synthesised, and an updated systematic review is overdue. The current protocol provides a description of the methods for conducting a systematic review aiming to synthesise the research concerning the effects of non-precarious employment on common mental health problems. Publishing a peer-reviewed protocol outlining the review methods before starting a review reduces the RoB due to ad hoc decisions during the review process [42]. To ensure a review of high quality, we will adhere to recommended guidelines for conducting and reporting systematic reviews.

A challenge when synthesising evidence from observational studies is the risk of non-reporting bias [43]. Preregistration and protocols are not mandatory for observational studies, making it difficult to assess publication bias within and across studies [43]. To limit this RoB as far as possible, we will scrutinise the included papers for signs of selective outcome reporting. Furthermore, we will conduct a comprehensive search for grey literature through OpenAlex, which indexes about 250,000 different sources.

The review described in the current protocol is an update of a systematic review published by van der Noordt and colleagues in 2014. We will expand the previous review by including studies published after 2012 and include grey literature. Additionally, we plan to conduct subgroup analyses to investigate possible mediation effects. Although challenging to study, knowledge about possible mental health benefits of employment is important for policy makers, researchers, and occupational health professionals. Therefore, the aim of the planned review is to synthesise the best evidence available about possible effects of non-precarious employment on common mental health problems.

### Supplementary Information

Additional file 1. PRISMA-P 2015 Checklist.

Additional file 2. Appendix 1 Search strategy.

## Data Availability

Not applicable.

## Abbreviations

BDI: Beck Depression Inventory
CES-D: Centre for Epidemiological Studies-Depression
CI: Confidence interval
COSMOS-E: Guidance on Conducting Systematic Reviews and Meta-analyses of Observational Studies of Etiology
DQ: Distress questionnaire
GAD: Generalised anxiety disorder
GHQ: General Health Questionnaire
HSCL: Hopkins Symptom Checklist
ILO: International Labour Organizations
MeSH: Medical Subject Headings
MHI: Mental Health Inventory
MOOSE: Proposal for reporting of Meta-analysis Of Observational Studies in Epidemiology
NOS: Newcastle-Ottawa scale
N-RCT: Non-randomised controlled trial
OR: Odds ratio
PHQ: Patient-reported health questionnaire
PRISMA: Preferred Reporting Items for Systematic review and Meta-Analysis
PRISMA-P: Preferred Reporting Items for Systematic review and Meta-Analysis Protocols
PROSPERO: International Prospective Register of Systematic Reviews
RCT: Randomised controlled trial
RevMan: Review Manager
RoB: Risk of bias
RoB 2: Cochrane risk-of-bias tool for randomised trials version 2
ROBINS-I: The Risk Of Bias In Non-randomised Studies of Interventions
SMD: Standardised mean difference
SWiM: Synthesis Without Meta-analysis in systematic reviews reporting guideline
