# Supplementary material for: Employment and mental health in the working age population: a protocol for a systematic review of longitudinal studies
Source: Syst Rev. 2024 Jul 25;13:197. doi: 10.1186/s13643-024-02613-1 (PMC11274751; doi:10.1186/s13643-024-02613-1)
Supplement: Supplementary file 2 — Additional file 2. Appendix 1 Search strategy. [file 13643_2024_2613_MOESM2_ESM.docx]

## Appendix 1, search strategy

[Ovid MEDLINE(R) ALL <1946 to March 21, 2023>](https://ovidsp.ovid.com/ovidweb.cgi?T=JS&NEWS=N&PAGE=main&SHAREDSEARCHID=18pCjXHf7wuXQV9hrgGAsSrhvHBqGiZ13hXVoDS9BjlN5wOXLoGsutp6Lwxfz23dd)

1 employment/ 50214

2 (work or working or worker or employee* or employment or employed or "labo?r participation" or job or re-employment or re-employed).ti,ab,kf,bt. 2029243

3 1 or 2 2046659

4 (unemployed or unemployment or non-employed or un-employed or un-employment or laid-off or workless or "out of work" or "out of job").ti,ab,kf,bt. 24482

5 unemployment/ 7750

6 4 or 5 27243

7 mental health/ or depression/ or anxiety/ or psychological distress/ or mental disorders/ or mentally ill persons/ 433903

8 ((psychological* or mental*) adj3 (health or distress or disorder* or ill*ness)).ti,ab,kf,bt. 328768

9 (depress* or anxiet*y).ti,ab,kf,bt. 680634

10 7 or 8 or 9 1056323

11 3 and 6 and 10 3652

12 11 and 2012:2023.(sa_year). 2376
